# Controllable Synthesis of Silicalite-1 with Tailored c-Axis Length via KHSO_4_ and Seed Co-Additive Strategy

**DOI:** 10.3390/ma19122634

**Published:** 2026-06-18

**Authors:** Xiaojing Meng, Liangxu Zhou, Junwei Huang, Min Li

**Affiliations:** College of Chemistry and Chemical Engineering, Chongqing University of Science and Technology, Chongqing 401331, China; 2023205045@cqust.edu.cn (L.Z.); 2025206058@cqust.edu.cn (J.H.);

**Keywords:** silicalite-1, seed crystal precursors, KHSO_4_, adsorption performance

## Abstract

Zeolite morphology strongly determines its performance. Herein, Silicalite-1 was synthesized in a low-template system (TPA^+^/Si = 0.007) via a synergistic strategy using potassium bisulfate and seed suspension. The seeds supply abundant structural units to reduce nucleation barrier and accelerate crystallization, while KHSO_4_ facilitates silicate polycondensation and suppresses non-MFI impurities. Sulfate ions selectively adsorb on specific crystal facets via hydrogen bonding and induce preferential crystal growth along the c-axis. The c-axis size of Silicalite-1 can be precisely regulated by adjusting dosages of seeds and KHSO_4_. Well-defined plate-like crystals were obtained under the conditions of K^+^/Si = 0.25, a seed content of 2.42 wt%, and hydrothermal treatment at 180 °C for 8 h. Scale-up synthesis in a 2 L autoclave verifies its industrial potential. The product exhibits excellent adsorption capacity and cyclic stability toward methylene blue. This work provides a low-cost and green route for morphology-controlled synthesis of MFI-type zeolites.

## 1. Introduction

Silicalite-1, an aluminum-free zeolite, possesses distinct hydrophobicity and chemical inertness, endowing it with considerable value in catalysis, adsorption separation, and membrane applications [1,2,3,4,5,6,7,8]. In traditional synthesis methods, organic templates can induce the nucleation and growth of crystals; however, they are accompanied by economic costs and environmental issues [9,10,11,12]. Consequently, reducing the consumption of templating agents or developing organic template-free alternatives has become an urgent research focus in sustainable synthesis.

In the template-free synthesis method, silicon species are spontaneously assembled into the MFI structure under the induction of inorganic ions. However, the directing effect of inorganic ions is weaker than that of organic templates, making it difficult to accurately match the pore size and charge distribution of the MFI structure, which easily leads to the formation of non-MFI impurity phases such as cristobalite [13]. Furthermore, the template-free method has issues that are unfavorable for industrial applications: it requires strict control over reaction parameters (e.g., alkalinity, crystallization temperature, etc.), and the resulting products exhibit irregular crystal morphology, wide particle size distribution, and poor dispersibility [14,15,16,17].

By contrast, the low organic template method (TPA^+^/Si < 0.01) is more economical and environmentally friendly owing to its low template dosage. This method usually combines the use of seeds and additives to offset the adverse effects of reduced template dosage on the crystallization process, thereby ensuring high crystallization efficiency and product purity. Seeds are typically employed to promote nucleation and crystallization, which are fully dispersed in the system to act as nucleation sites, lowering the activation energy required for nucleation and further inducing the growth of new nuclei in the system. However, seeds cannot avoid the formation of the quartz phase. Furthermore, the dispersion degree of powdered seeds also affects the properties of the product; insufficient dispersion will lead to severe agglomeration and larger particle size of the product [18,19,20,21,22]. The use of seed suspension successfully addresses this issue, which not only enhances its dispersion in the system and improves the uniformity of structural units, but also renders the nucleation process more controllable. Nevertheless, seed suspension still fails to inhibit the formation of the quartz phase [23,24].

The type of silicon source plays a critical role in influencing the crystallization behavior and product quality of Silicalite-1 zeolite, with this effect being particularly pronounced in low-template systems. Commonly employed silicon sources include tetraethyl orthosilicate (TEOS), silica gel, and fumed silica. TEOS exhibits a moderate and homogeneous hydrolysis rate, facilitating the formation of crystals with small particle sizes, excellent dispersity, and low impurity content [23]. However, its high cost limits practical industrial applications. Fumed silica features high purity but is prone to particle agglomeration and requires strongly alkaline conditions for complete dissolution, which not only broadens the particle size distribution of the product but also increases the likelihood of forming impurity phases such as quartz and cristobalite [25]. In comparison, silica gel offers a favorable balance between reactivity and production cost. Moreover, its moderate hydrolysis and depolymerization kinetics enable a sustained and steady supply of silicate species, effectively suppressing the formation of non-MFI impurity crystals and further improving crystal uniformity [24].

To suppress the formation of non-zeolitic phases and achieve precise regulation of the crystal morphology, pore structure, and performance of zeolites, researchers have introduced additives along with seed crystals during the zeolite synthesis process. Currently reported additives can be roughly categorized into three classes: fluorides, alcohols, and amino-containing compounds (e.g., urea, tetramethylguanidine). A comprehensive analysis of the additive’s role reveals the following: ① Fluorides [19,25,26,27] (e.g., NH_4_F, KF, NaF) can act as mineralizers, in which F^−^ forms stable Si-F bonds (such as SiF_6_^2−^) with silicon species, promoting the slow depolymerization and rearrangement of silicon sources over a wide pH range, which effectively reduces the activation energy required for crystallization. In addition, F^−^ can be selectively adsorbed on specific crystal planes ([010] and [100] planes) of Silicalite-1 crystals, inhibiting crystal growth in these directions. Meanwhile, it reduces the hydrogen bonding interaction between crystals, preventing the formation of large agglomerates and ultimately yielding crystals with better dispersibility. Nevertheless, the extensive use of fluorides can cause equipment corrosion and environmental pollution. ② Alcohols [28,29,30] (e.g., ethanol, propanol) are amphiphilic and can be adsorbed on the surface of silicon source particles or initial crystal nuclei, which can reduce the solid–liquid interfacial tension and prevent particles from agglomerating due to van der Waals forces, thereby improving the solubility of silicon sources and avoiding disordered aggregation caused by excessively high local concentrations of silicon sources—laying a foundation for subsequent uniform crystallization. Moreover, alcohols can form hydrogen bonds or hydrophobic interactions with templates (e.g., TPABr, TPAOH), enhancing the adsorption selectivity of templates on specific crystal planes (such as the [100] plane of MFI structure) and guiding crystal growth along specific directions (regular cubes, short columns, or thin sheets). Still, the high interaction energy associated with alcohols prolongs the crystallization process. ③ Amino-containing compounds [23,24,31] undergo gradual hydrolysis to generate NH_3_·H_2_O under heating conditions (typically 60~120 °C), which further dissociates to release OH^−^, resulting in a slow rather than sudden increase in the alkalinity of the system. This “slow-release” property prevents the rapid hydrolysis and agglomeration of silicon sources caused by excessively high local OH^−^ concentration, providing a stable alkaline environment for the uniform growth of Silicalite-1 crystals. The amino groups (-NH_2_) produced by urea hydrolysis can be adsorbed on the surface of silicon source particles or initial crystal nuclei, which prevent direct contact between particles through a steric hindrance effect, reducing crystal agglomeration and facilitating the formation of products with better dispersibility. However, the requirement for OH^−^ generated via hydrolysis prolongs the time needed for the system to reach the required alkalinity, making the crystallization cycle of the urea system longer than that of the direct alkali-adding system. Furthermore, the hydrolysis rate of urea is strictly dependent on temperature: excessively low temperatures lead to slow hydrolysis and difficulty in initiating crystallization; excessively high temperatures may cause rapid decomposition of urea, resulting in a sudden increase in OH^−^. This, in turn, triggers the agglomeration of silicon sources, necessitating precise control of the reaction temperature and indirectly increasing the difficulty of process control.

A comprehensive analysis of the roles of the three types of additives reveals that, except for F^−^ which can directly form Si-F bonds with silicon species, alcohols and amino groups mainly regulate crystal growth through hydrogen bonding interactions between silanol groups, or between amino groups and silanol groups [30]. Therefore, it may be feasible to replace alcohols or amino-containing compounds with other substances capable of forming hydrogen bonds, thereby addressing the problems caused by their strong interaction or slow alkali release. Researchers introduced sodium sulfate (Na_2_SO_4_) into a template-free system, confirming that the interaction between sulfate ions (SO_4_^2−^) and silanol groups can accelerate the polycondensation of silicates, thus successfully synthesizing ZSM-5 molecular sieves. However, this method still requires a relatively long crystallization time (24 h) under template-free conditions and necessitates the use of sulfuric acid (H_2_SO_4_) to adjust the system pH, which not only increases additional costs but also introduces operational risks [32]. Based on the aforementioned reports, we propose that potassium bisulfate (KHSO_4_) is expected to serve as a viable alternative additive: potassium ions have been proven to be excellent pore-forming agents that can regulate the pore structure of zeolites [25]; sulfate ions can promote the polycondensation of silicon sources, while the released hydrogen ions can directly adjust the system pH without the need for additional acid sources.

In this study, Silicalite-1 zeolites were synthesized by using the combination of the seed precursor and KHSO_4_ in a very low organic template system (TPA^+^/Si = 0.007). The mechanism of action of additives in the synthesis process was discussed, and a reasonable crystallization mechanism was proposed. In addition, scale-up synthesis of the zeolite was successfully realized in a 2 L autoclave under the optimized experimental conditions. Adsorption tests verified that the as-synthesized zeolite exhibits excellent purification performance for methylene blue wastewater. This work may provide a new avenue for large-scale and low-cost production of high-quality zeolite for industrial applications.

## 2. Materials and Methods

### 2.1. Materials

The synthesis of the seed crystal-directed solution and the product zeolite was carried out using silica gel (SiO_2_, 40%, Qingdao Maclean Silica Gel Drying Agent Co., Ltd., Qingdao, China), tetrapropylammonium bromide (TPABr, 98%, Maclean Biochemical Technology Co., Ltd., Shanghai, China), sodium hydroxide (NaOH, AR, Chengdu Kelong Chemical Co., Ltd., Chengdu, China), and potassium bisulfate (KHSO_4_, AR, 98%, Maclean Biochemical Technology Co., Ltd.). Deionized water (H_2_O) was prepared in the laboratory. All reagents were used as received without further purification.

### 2.2. Methods

#### 2.2.1. Preparation of Seed Crystal Precursor (Hereinafter Referred to as Seeds)

The molar composition is 1SiO_2_:0.056TPABr:0.24NaOH:13.64H_2_O. First, 36.7 g of water was added into a beaker, followed by the addition of 26.5 g of silica gel and 2.67 g of tetrapropylammonium bromide (TPABr). The mixture was stirred at room temperature for 10 min to obtain solution A. Separately, 1.67 g of sodium hydroxide was dissolved in 6.67 g of water, and the solution was cooled to room temperature to give solution B. Under continuous stirring, solution B was slowly added dropwise to solution A to form a synthesis gel. The gel was stirred at room temperature for 3 h, then transferred into a Teflon-lined stainless steel autoclave and heated at 80 °C for 16 h to obtain a seed suspension.

#### 2.2.2. Synthesis of Silicalite-1

The initial molar composition of Silicalite-1 was 1SiO_2_:0.007TPABr:x KHSO_4_:13.64H_2_O:y NaOH:z Seeds. First, 36.7 g of water was added into a beaker, followed by the addition of 26.5 g of silica gel and 0.33 g of tetrapropylammonium bromide (TPABr). The mixture was stirred at room temperature for 10 min. Then, seed crystals and potassium bisulfate were added, and stirring was continued for another 10 min to obtain solution C. Separately, 1.67 g of sodium hydroxide was dissolved in 6.67 g of water, and the solution was cooled to room temperature to give solution D. Under continuous stirring, solution D was slowly added dropwise to solution C to form a synthesis gel. The gel was stirred at room temperature for 3 h, then transferred into a Teflon-lined stainless steel autoclave and heated at 180 °C for 8 h. After the reaction, the solid product was collected by filtration, washed with deionized water until neutral, then dried at 120 °C for 3 h, and finally calcined at 550 °C for 5 h to remove the template.

The resulting samples were denoted as S1-x-y-z, where x is the molar ratio of potassium ions to the silica source (x = 0–0.25), y is the molar ratio of sodium ions to the silica source (y = 0.19–0.29), and z is the mass fraction of seed crystals in the synthesis gel (z = 0–4.21 wt%).

#### 2.2.3. Scale-Up Experiment

Detailed experimental procedures for the scale-up design are provided in the Supplementary Materials.

#### 2.2.4. Synthesis with Other Additives

Detailed experimental procedures for the synthesis with other additives are available in the Supplementary Materials.

#### 2.2.5. Adsorption Test for Methylene Blue Wastewater

Detailed experimental procedures for the adsorption tests are provided in the Supplementary Materials.

### 2.3. Characterization

Phase analysis of Silicalite-1 was performed using a Rigaku SmartLab-9 diffractometer (XRD, Shimadzu XRD-6000, Shimadzu, Tokyo, Japan, Cu-Kα radiation, λ = 1.5418 Å, 40 kV, 100 mA). The scan rate was set at 8°/min and the scan 2θ angle range was 5 to 60°. Crystallinity was defined as the sum of diffraction peak intensities at the five main peaks at 2θ = 7.9°, 8.9°, 23.1°, 23.9° and 24.4°. The crystallinity of sample S1-0.1-0.24-4.21 wt% was taken as the reference (*R*_1_) and set to 100.00%. The relative crystallinity (RC) was calculated as the ratio of the crystallinity of other samples (*R*_2_) to that of the reference sample (*R*_1_), and the corresponding formula is shown in Equation (1).

(1) $$RC=\frac{R_{2}}{R_{1}}\times 100\%$$

*R*_2_ is for the synthesized samples, and *R*_1_ is for S1-0.1-0.24-4.21 wt%.

In addition, the crystallinity of silica was defined as the sum of diffraction peak intensities at 2θ = 5.7°, 25.78°, 26.94° and 28.2°. Sample S1-0-0.24-0 was selected as the reference, with its silica crystallinity (*SR*_1_) set to 100.00%. The relative crystallinity of silica (SRV) was calculated as the ratio of the silica crystallinity of other samples (*SR*_2_) to (*SR*_1_). The calculation formula for SRV is presented in Equation (2).

(2) $$SRV=\frac{{SR}_{2}}{{SR}_{1}}\times 100\%$$

*SR*_2_ is for the synthesized samples, and *SR*_1_ is for S1-0-0.24-0.

Scanning electron microscopy (SEM) combined with energy dispersive spectroscopy (EDS) was used to characterize the morphology and elemental composition of the samples. The test was performed on a JEOL JSM-7800F field emission scanning electron microscope (FE-SEM, JEOL Ltd., Tokyo, Japan). All samples were coated with gold before measurement, and the accelerating voltage was set at 5 kV.

Pore structure parameters were acquired using a 3Flex 5.02 adsorption analyzer (Micromeritics Instrument Corporation, Norcross, GA, USA). The specific surface area, pore volume and other related data were calculated based on different theoretical models.

The skeleton vibration of the zeolite was detected by the NexusTM-type infrared spectroscopy Fourier transform infrared spectrometer (FT-IR, Thermo Nicolet Corporation, Madison, WI, USA). The skeleton vibration of the zeolite was performed by potassium bromide tableting, and with the blank potassium bromide tablets as the background, the DTGS detector was used; the number of acquisitions was 32, and the resolution was 4.

The residual concentration of methylene blue in solution was determined using a T6 New Century UV-Vis spectrophotometer (Beijing Persee General Instrument Co., Ltd., Beijing, China) at 664 nm. The absorbance was proportional to the concentration of methylene blue.

## 3. Results and Discussion

### 3.1. The Role of Seed

The state of the seed suspension significantly affects the deduction of the crystallization mechanism. As shown in Figure S1a, the seed suspension prepared in this work possesses no crystalline structure and consists of amorphous silicate species. In the FT-IR spectrum, only the bending vibration peak of Si-O bonds at 453 cm^−1^, the symmetric stretching vibration peak of Si-O-Si bonds at 790 cm^−1^, and the asymmetric stretching vibration peaks at 1120 cm^−1^ and 1220 cm^−1^ were observed [24]. No characteristic peak corresponding to the double five-membered ring structure at 549 cm^−1^ was detected (Figure S1b). This result indicates that the seed suspension is an amorphous gel composed of abundant structural units.

A series of experiments were conducted to investigate the effect of seeds on the synthesis of Silicalite-1 zeolite under the gel composition of 1 SiO_2_:0.007 TPABr:0.24 NaOH:13.66 H_2_O:z seeds (z = 0~4.21 wt%).

**Figure 1:**
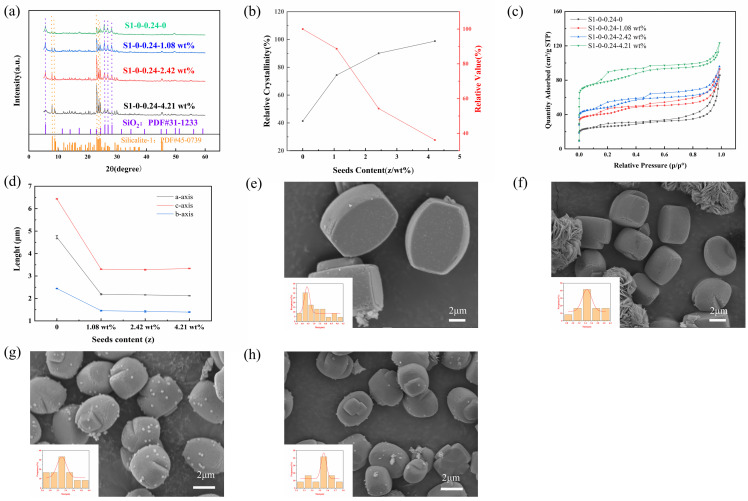
Silicalite-1 with different seed contents: (**a**) XRD; (**b**) RC and SRV; (**c**) N_2_ adsorption–desorption isotherms; and (**d**) the length along the three axes. SEM images of (**e**) S1-0-0.24-0; (**f**) S1-0-0.24-1.08 wt%; (**g**) S1-0-0.24-2.42 wt%; and (**h**) S1-0-0.24-4.21 wt%.

The XRD patterns, N_2_ adsorption–desorption isotherms, and SEM images of the samples are shown in Figure 1. All samples exhibited strong characteristic peaks belonging to the MFI structure at 2θ = 7.9, 8.9, 23.1, 23.9, 24.4°. With increasing loading of seed crystal precursors, the relative crystallinity (RC) rose from 41.36% to 98.81%, accompanied by obvious improvement in phase purity. This phenomenon was attributed to the fact that seed crystal precursors lowered the nucleation barrier of zeolite and facilitate crystallization (Figure 1a).

Seed addition also exerts remarkable effects on crystal morphology and particle size. As presented in Figure 1e–h, the introduction of seed crystals changes the crystal morphology from the coexistence of twin crystals and cuboid particles (Sample S1-0-0.24-0, Figure 1e) to sole twin crystals (Sample S1-0-0.24-4.21 wt%, Figure 1h), with the crystal size decreasing from 6.5 μm to 2.7 μm. Specifically, crystal dimensions (Figure 1d) decreased from 4.75 μm to 2.12 μm along the a-axis, from 2.44 μm to 1.41 μm along the b-axis, and from 6.44 μm to 3.32 μm along the c-axis. This phenomenon originates from the abundant nucleation sites supplied by added seeds, which lower the nucleation energy barrier and accelerate the nucleation rate far beyond the crystal growth rate, consequently refining the crystalline grains. Moreover, synchronous shrinkage along all three crystallographic axes generates products with uniform particle size distribution and free of preferred orientation, verifying the absence of preferential facet growth throughout seed-mediated crystallization.

The reduction in crystal size generated more intercrystalline pores, resulting in an increase in the specific surface area (S_BET_) from 98.84 m^2^/g to 302.30 m^2^/g and an increase in the total pore volume (V_total_) from 0.13 cm^3^/g to 0.19 cm^3^/g (Table 1), which indicate that the addition of seed crystals provides nuclei, reduces the activation energy required for nucleation, induces crystal growth in the system [27], and enhances porosity. However, as observed in Figure 1a, all samples showed diffraction peaks belonging to SiO_2_ at 2θ = (5.7, 25.78, 26.94, 28.2°). Increasing the seed dosage can weaken the diffraction peak intensity of silica and reduce the crystallinity of SiO_2_ (SRV) from 100.00% to 36.17% (Table 1), which drives the transformation of silicon species toward the MFI framework. Nevertheless, the silica impurity phase cannot be entirely removed, and seed addition fails to thoroughly suppress the formation of miscellaneous crystalline phases.

To further investigate the role of seeds in the growth process of Silicalite-1, the gel composition was maintained as 1SiO_2_:0.007TPABr:0.24NaOH:13.66H_2_O:2.42 wt% seeds. Samples were taken at different time points.

**Figure 2:**
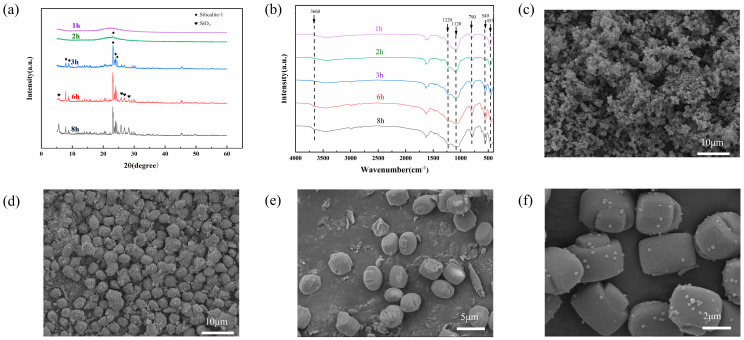
Synthesis products with different reaction times under 2.42 wt% seed system: (**a**) XRD; (**b**) FT-IR; SEM of (**c**) 2 h; (**d**) 3 h; (**e**) 6 h; (**f**) 8 h.

The XRD patterns, Fourier transform infrared (FT-IR) spectra, and SEM images of the samples are shown in Figure 2. When the crystallization time was 2 h, the sample was amorphous (Figure 2a). Only the absorption peaks at 453 cm^−1^ (bending vibration of Si-O bonds), 790 cm^−1^ (symmetric stretching vibration of Si-O-Si bonds), and 1120 cm^−1^ (asymmetric stretching vibration of Si-O-Si bonds) were observed in the FT-IR spectrum [24]. However, the characteristic peak at 549 cm^−1^ attributed to the double-five-ring structure was absent (Figure 2b), which indicates that although a large number of structural units were present in the system, the MFI structure had not been assembled. The presence of abundant flocculent substances and the absence of crystals in the SEM image (Figure 2c) further confirmed this inference. After 3 h of crystallization, five characteristic peaks belonging to the MFI structure appeared and were very intense, suggesting that the activated structural units rapidly assembled into the MFI crystal structure between 2 and 3 h (Figure 2a). The emergence of the characteristic peak at 549 cm^−1^ corresponding to the double-five-ring structure in the FT-IR spectrum, verified the formation of the MFI structure (Figure 2b). Meanwhile, a large number of crystal grains were observed in the SEM image (Figure 2d), and the grain surfaces were covered with numerous nanoparticles composed of silicon species. When the crystallization time reached at 6 h, the diffraction peaks of MFI were the strongest, indicating that the crystallization process was essentially complete (Figure 2a). In the FT-IR spectrum, the characteristic peaks became sharper and more intense, suggesting that the crystal growth was approaching thermodynamic equilibrium, the silicate framework was highly ordered, and crystallization was stabilized (Figure 2b). The number of surface nanoparticles decreased, and the corresponding silicon species participated in the crystallization process, further optimizing and perfecting the crystal structure (Figure 2e). When crystallization was prolonged to 8 h, some of the crystals dissolved and transformed into the more stable α-SiO_2_ structure, leading to a decrease in the intensity of the MFI diffraction peaks and an increase in the characteristic peaks of SiO_2_ (Figure 2a). Correspondingly, the intensities of the five infrared absorption peaks of the MFI structure also slightly decreased, which was consistent with the XRD results. Merely trace amounts of particles remained attached to crystal surfaces, marking the near-completion of crystallization.

### 3.2. The Role of KHSO_4_

The experiments were conducted to investigate the effect of KHSO_4_ alone on the synthesis of Silicalite-1 zeolite. The experiments were performed under the gel composition of 1SiO_2_:0.007TPABr:x KHSO_4_:0.24NaOH:13.66H_2_O (x = 0~0.15).

**Figure 3:**
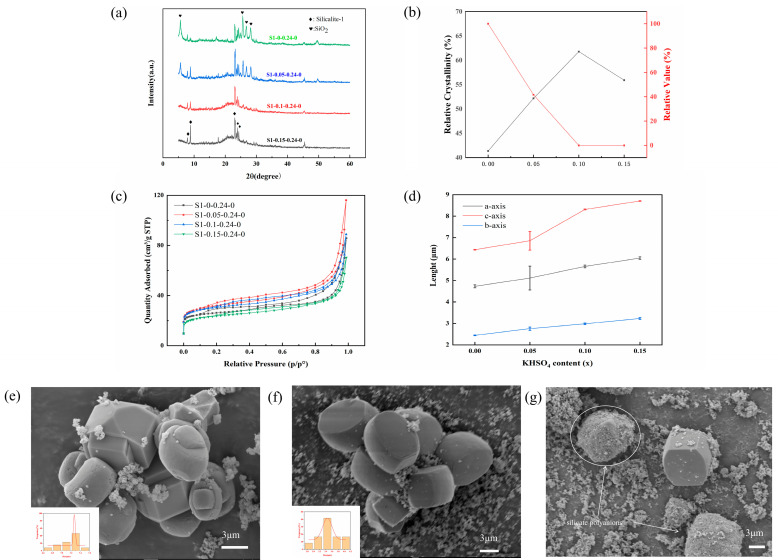
Silicalite-1 with different KHSO_4_ contents: (**a**) XRD; (**b**) RC and SRV; (**c**) N_2_ adsorption–desorption isotherms; and (**d**) the length along the three axes. SEM images of (**e**) S1-0.05-0.24-0; (**f**) S1-0.1-0.24-0; and (**g**) S1-0.15-0.24-0.

The XRD patterns, N_2_ adsorption–desorption isotherms, and SEM images of the samples are shown in Figure 3. All samples exhibited strong characteristic peaks of the MFI structure at 2θ = 7.9, 8.9, 23.1, 23.9, 24.4°, and the RC showed little change with the increase of KHSO_4_ (Figure 3a), indicating that KHSO_4_ has a minor contribution in promoting crystallization. Interestingly, KHSO_4_ is crucial in suppressing the formation of SiO_2_. When K^+^/Si reaches 0.1, the characteristic diffraction peaks of SiO_2_ are undetectable (SRV = 0) in the XRD pattern (Figure 3a). When K^+^/Si reaches 0.15, the RC of the sample decreases (Table 1), presumably because the addition of excessive H^+^ into the system prevents the gel from maintaining sufficient alkalinity to support the reaction. Additionally, crystal growth along the a-axis, b-axis, and c-axis was investigated (Figure 3d). Specifically, crystal dimensions increased from 4.75 μm to 6.07 μm along the a-axis, from 2.44 μm to 3.19 μm along the b-axis, and from 6.44 μm to 8.71 μm along the c-axis. The above results indicate that increasing the dosage of potassium bisulfate promotes crystal growth along all three crystallographic axes, with a slightly more significant increase observed along the c-axis. It is speculated that potassium bisulfate can induce preferential crystal growth. However, the narrow synthesis window restricts further addition of this additive.

Compared with the S1-0-0.24-0 sample, in the presence of KHSO_4_ (Figure 3e–g), the crystal particles exist in an aggregated state, and the grain size increases from 6.44 μm to 8.71 μm. The aggregated crystals generate additional intercrystalline pores. Therefore, the specific surface areas with KHSO_4_ added (S1-0.05-0.24-0 and S1-0.1-0.24-0) are higher than that of S1-0-0.24-0, while the addition of excessively high amount of H^+^ affects crystallization led to the decrease in the specific surface area (S1-0.15-0.24-0) (Table 1).

Overall, potassium bisulfate can remarkably inhibit the formation of silica. The optimal molar ratio of K^+^/Si = 0.1. A lower ratio fails to suppress the generation of silica impurities, while an excessive dosage reduces the system pH and impairs crystallization. In addition, KHSO_4_ also exerts an influence on crystal growth orientation, yet this effect cannot be fully manifested due to the narrow synthesis window, which requires the assistance of seed crystals.

Considering that pH directly affects the aggregation state of silicon species, nucleation rate, crystallization time, gel stability, and the aqueous solution of KHSO_4_ can change the pH of the system, it is necessary to investigate the influence of alkalinity. The experiments were carried out in a gel system of 1SiO_2_:0.007TPABr:0.1KHSO_4_:y NaOH:13.66H_2_O (y = 0.19~0.24).

**Figure 4:**
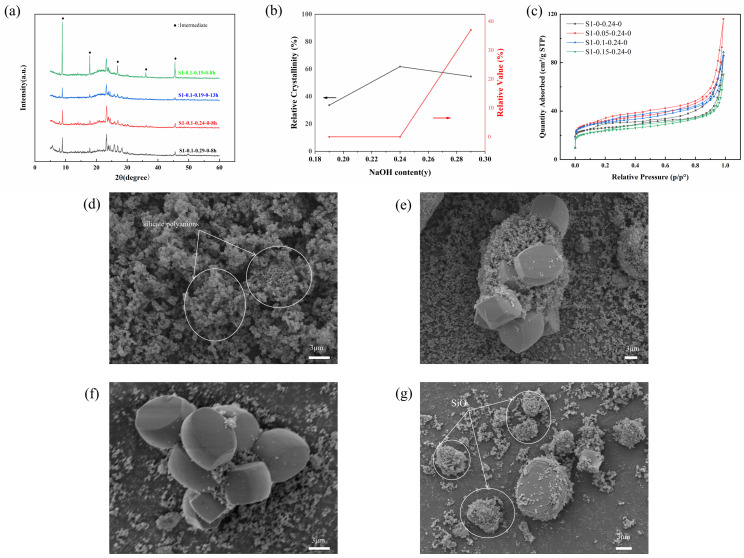
Silicalite-1 with different NaOH contents: (**a**) XRD; (**b**) RC and SRV. (**c**) N_2_ adsorption–desorption isotherms and SEM images of (**d**) S1-0.1-0.19-0 (8 h); (**e**) S1-0.1-0.19-0 (13 h); (**f**) S1-0.1-0.24-0; and (**g**) S1-0.1-0.29-0.

Figure 4 presents XRD patterns, N_2_ adsorption–desorption isotherms, and SEM images. At y = 0.19, crystallization remained incomplete after 8 h, with non-MFI diffraction peaks appearing alongside MFI reflections. Extending crystallization to 13 h eliminated these extraneous peaks while intensifying MFI signals (Figure 4a). Combined with abundant amorphous flocculent material observed in Figure 4d, these peaks likely correspond to intermediate crystals formed during silicon species transformation to the MFI structure mediated by KHSO_4_. Increasing alkalinity to y = 0.24 enabled complete crystallization within 8 h. No SiO_2_ diffraction peaks appeared in this sample (SRV = 0, Figure 4a, Table 1). Sulfate ions (SO_4_^2−^) promote silicate polycondensation into polyanions [30], which subsequently convert to zeolites under template guidance. This mechanism explains KHSO_4_’s inhibition of SiO_2_ crystalline phase formation. However, high H^+^ concentration prolongs crystallization and slows the intermediate-to-MFI transformation, causing intermediate phase diffraction peaks in S1-0.1-0.19-0-8 h (Figure 4a). The crystallization mechanism proceeds as follows: KHSO_4_ addition promotes silicate polycondensation into polyanions. These polyanions form intermediate crystals at elevated temperatures before converting to MFI-structured zeolites under structure-directing agent induction. At y = 0.29, characteristic SiO_2_ diffraction peaks reappear (2θ = 5.7°, 25.78°, 26.94°, 28.2°; Figure 4a), accompanied by flake-composed silicon dioxide spheres (Figure 4g). This suggests excessive alkalinity compromises SO_4_^2−^’s inhibitory effect. Potassium salts serve as effective pore-forming agents [25], with S_BET_ and V_total_ values increasing concomitantly with alkalinity (Table 1).

To further investigate the effect of KHSO_4_ on the synthesis of Silicalite-1 zeolite, the gel composition was maintained as 1SiO_2_:0.007TPABr:0.1KHSO_4_:0.24NaOH:13.66H_2_O, and static heating was performed at 180 °C. Samples were taken at different time points.

**Figure 5:**
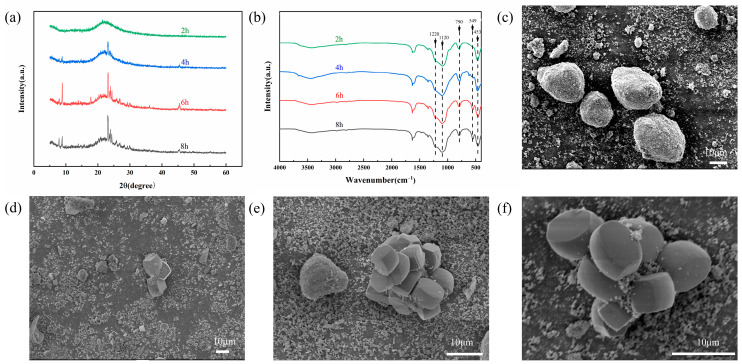
Synthesis products with different reaction times under KHSO_4_ alone system: (**a**) XRD; (**b**) FT-IR; SEM of (**c**) 2 h; (**d**) 4 h; (**e**) 6 h; and (**f**) 8 h.

Figure 5 presents XRD patterns, FT-IR spectra, and SEM images of the samples. Weak MFI characteristic diffraction peaks (2θ = 7.9°, 8.9°, 23.1°, 23.9°, 24.4°; Figure 5a) emerged at 4 h, indicating MFI framework formation and crystal growth initiation following a 4 h induction period. At 6 h, faint non-MFI diffraction peaks appeared, resembling the XRD pattern of S1-0.1-0.19-0 at 8 h (Figure 4a) and corresponding to the intermediate crystalline phase during silicate polyanion transformation. Subsequently, MFI peak intensity progressively increased while the intermediate phase diminished, suggesting crystallization proceeded to completion. SEM images revealed spherical silicate polyanion particles exceeding 20 μm diameter at 2 h (Figure 5c). Initial crystals appeared at 4 h (Figure 5d), evolving into aggregated Silicalite-1 crystals by 6 h and 8 h. FT-IR analysis (Figure 5b) further elucidated crystallization. At 2 h, peaks at 453 cm^−1^ (Si-O bending), 790 cm^−1^ (Si-O-Si symmetric stretch), and 1120 cm^−1^ (Si-O-Si asymmetric stretch) confirmed amorphous silicate species with disordered frameworks. The absence of the 549 cm^−1^ peak (double five-membered rings) supported this. By 4 h, the emergence of the 549 cm^−1^ peak indicated ordered aggregation of silicon species. At 6 h, a new peak at 1220 cm^−1^ (pore-sensitive asymmetric stretch) appeared alongside sharpened peaks at 549 cm^−1^ and 790 cm^−1^, signifying formation of two-dimensional 10-membered ring channels alongside increased crystallinity and order. Finally, maximum-intensity narrow and strong peaks at 8 h confirmed complete crystal growth with a highly ordered silicate framework. It is worth noting that, compared with the FT-IR spectrum in the presence of seed crystals alone (Figure 2b), the characteristic peak at 3660 cm^−1^ attributed to isolated silanol groups is not observed in Figure 5b. Correspondingly, the weak and broad peak in the range of 3300–3600 cm^−1^ in Figure 5b is stronger than that in Figure 2b. This phenomenon is caused by the red shift of O–H bonds induced by the hydrogen bonding interaction between sulfate ions and silanol groups [33].

Based on the experimental results, the crystallization process is proposed as follows. After adding KHSO_4_, sulfate ions (SO_4_^2−^) promote polycondensation of the silicon source into silicate polyanions [32]. These polyanions aggregate into spherical structures, within which crystal nuclei form. The silicate polyanions initially generate an intermediate crystalline phase, subsequently transforming into Silicalite-1 zeolite under the structure-directing agent. This mechanism explains the aggregated crystal distribution observed with KHSO_4_: silicate anions aggregate into spheres, confining nucleation and crystal growth within these domains. Once the silicate polyanions are consumed, the resulting crystals remain aggregated. The sample S1-0.1-0.19-0 exhibited an intense intermediate crystalline phase XRD peak at 8 h (Figure 4a), whereas this peak remained very weak throughout the crystallization of S1-0.1-0.24-0 (Figure 5a). This difference arises because silicate polyanions firstly form an intermediate phase, which then converts to the MFI-type zeolite. Lower alkalinity (y = 0.19) slows the transformation rate of the intermediate phase to the MFI structure, causing intermediate phase accumulation and intense XRD peaks. Conversely, higher alkalinity (y = 0.24) accelerates the transformation, preventing significant intermediate phase accumulation and resulting in extremely weak XRD peaks for this phase.

### 3.3. Synergistic Effect of KHSO_4_ and Seeds

The above experimental results show that KHSO_4_ can effectively inhibit the formation of SiO_2_, and seeds can act as existing crystal nuclei to reduce the activation energy for nucleation, thereby inducing crystal growth in the system. Therefore, the synergistic effect of KHSO_4_ and seeds is expected to produce zeolites with high crystallinity and pure phase.

#### 3.3.1. Effect of Seeds in the Presence of KHSO_4_

To investigate the effect of seeds on the synthesis of Silicalite-1 zeolite in the presence of KHSO_4_, experiments were conducted with a gel composition of 1SiO_2_:0.007TPABr:0.1KHSO_4_:0.24NaOH:13.66H_2_O:z seeds (z = 0~4.21 wt%).

**Figure 6:**
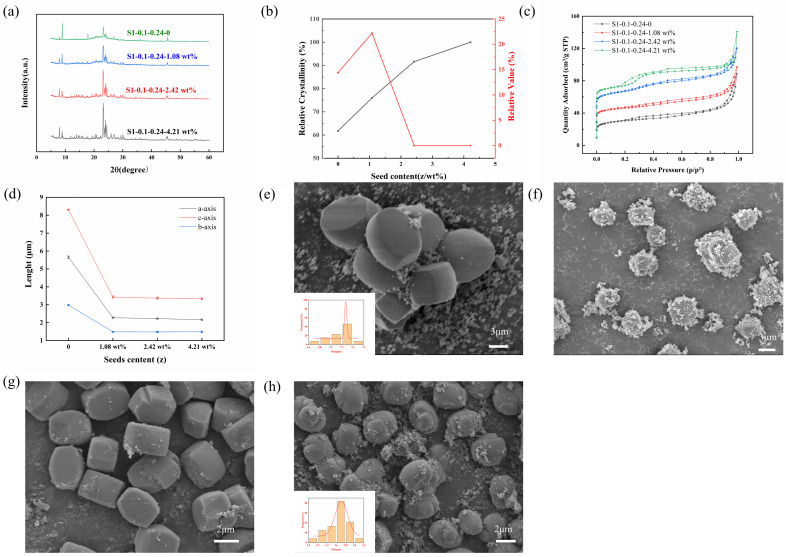
K^+^/Si = 0.1 with different seed contents: (**a**) XRD; (**b**) RC and SRV; (**c**) N_2_ adsorption–desorption isotherms; and (**d**) the length along the three axes. SEM images of (**e**) S1-0.1-0.24-0; (**f**) S1-0.1-0.24-1.08 wt%; (**g**) S1-0.1-0.24-2.42 wt%; and (**h**) S1-0.1-0.24-4.21 wt%.

The XRD patterns, N_2_ adsorption–desorption isotherms, and SEM images of the samples are shown in Figure 6. All samples exhibited strong characteristic peaks of the MFI structure, and the intensity of the diffraction peaks increased with the increase in seed addition (Figure 6a), demonstrating the role of seeds. Moreover, the crystallinity (RC) of the samples containing KHSO_4_ (S1-0.1-0.24-1.08 wt% (RC = 76.00%), S1-0.1-0.24-2.42 wt% (RC = 91.63%)) was slightly higher than that of the equivalent samples without KHSO_4_ (S1-0-0.24-1.08 wt% (RC = 74.43%), S1-0-0.24-2.42 wt% (RC = 90.09%)) (Table 1). Furthermore, the characteristic peaks of SiO_2_ were only observed in the sample S1-0.1-0.24-1.08 wt%, and its SRV was much lower than that of S1-0-0.24-1.08 wt% (Table 1), this indicates that the effects of seeds and KHSO_4_ are not independent, and their synergistic interaction facilitated the synthesis of zeolites with high crystallinity and purity. With the continuous addition of seeds, the grain size decreased from 8.3 μm (S1-0.1-0.24-0) to 3.5 μm (S1-0.1-0.24-4.21 wt%), which generated more intercrystalline pores and subsequently increased the specific surface area (Table 1). However, the presence of KHSO_4_ made the size of S1-0.1-0.24-4.21 wt% (3.5 μm) slightly larger than that of S1-0-0.24-4.21 wt% (2.7 μm). The crystal morphology changed from aggregated tetragonal forms (Figure 6e) to uniformly distributed weight-like forms (Figure 6h), because excessive seeds disrupted the aggregation of silicate polyanions into spheres, and the dispersed crystal nuclei in the system prevented the growth of grains from piling up. Additionally, the crystal dimensions decreased from 5.67 μm to 2.16 μm along the a-axis, from 2.98 μm to 1.49 μm along the b-axis, and from 8.32 μm to 3.33 μm along the c-axis, which demonstrates that even in the presence of KHSO_4_, increasing seed content induces synchronous reduction along all three crystallographic axes, yielding uniform size distributions. KHSO_4_ preserves seed function, and additional seeds exhibit no facet-specific selectivity during crystal growth.

In terms of he pore structure, the specific surface areas of S1-0.1-0.24-1.08 wt% (179.89 m^2^/g) and S1-0.1-0.24-2.42 wt% (258.53 m^2^/g) are slightly higher than those of S1-0-0.24-1.08 wt% (157.37 m^2^/g) and S1-0-0.24-2.42 wt% (186.10 m^2^/g) (Table 1), which is attributed to the presence of KHSO_4_. The silicate polyanions induce grain stacking, which generates additional intercrystalline pores, thereby resulting in the higher specific surface areas. However, when the seed content reaches 4.21 wt%, the seeds become the dominant factor in the system, and the pore structure is controlled by the seeds. As a result, the specific surface areas of S1-0.1-0.24-4.21 wt% (299.74 m^2^/g) and S1-0-0.24-4.21 wt% (302.30 m^2^/g) are comparable (Table 1).

#### 3.3.2. Effect of KHSO_4_ in Presence of Seeds

To investigate the effect of KHSO_4_ on the synthesis of Silicalite-1 in the presence of seeds, experiments were conducted under a gel composition of 1SiO_2_:0.007TPABr:xKHSO_4_:0.24NaOH:13.66H_2_O:2.41wt% seeds (x = 0~0.25).

**Figure 7:**
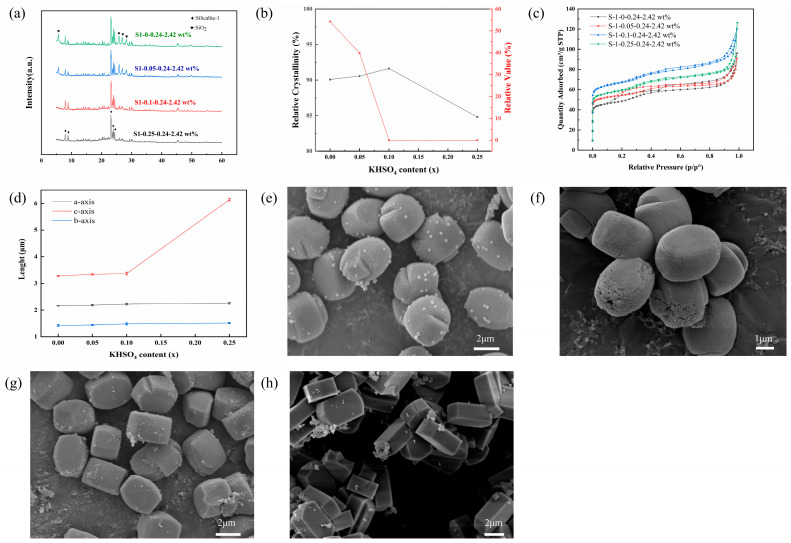
Seed = 2.42 wt% with different KHSO4 contents: (**a**) XRD; (**b**) RC and SRV; (**c**) N_2_ adsorption–desorption isotherms; and (**d**) the length along the three axes. SEM images of (**e**) S1-0-0.24-2.42 wt%; (**f**) S1-0.05-0.24-2.42 wt%; (**g**) S1-0.1-0.24-2.42 wt%; and (**h**) S1-0.25-0.24-2.42 wt%.

The XRD patterns, N_2_ adsorption–desorption isotherms, and SEM images of the samples are shown in Figure 7. All samples exhibited strong characteristic peaks of the MFI structure at 2θ = 7.9, 8.9, 23.1, 23.9, 24.4°. Consistent with expectations, when K^+^/Si reached 0.1, the characteristic peaks of SiO_2_ (2θ = 5.7, 25.78, 26.94, 28.2°) disappeared, demonstrating the ability of KHSO_4_ to inhibit SiO_2_ (Figure 7a). Meanwhile, KHSO_4_ exerts a slight promoting effect on crystallization, leading in the gradual increase in relative crystallinity (RC) of S1-0-0.24-2.42 wt% (RC = 90.09%), S1-0.05-0.24-2.42 wt% (RC = 90.56%), and S1-0.1-0.24-2.41 wt% (RC = 91.63%). However, when the content of KHSO_4_ was too high (x = 0.25), the addition of excessive H^+^ changed the pH of the system, and the RC value decreased to 180.84%. Interestingly, the addition of KHSO_4_ also changed the crystal morphology from uniformly distributed weight-like crystals (Figure 7d) to aggregated sheet-like crystals with a b-axis thickness of 1.55 μm (Figure 7h). The crystal dimensions in Figure 7d increased from 2.16 μm to 2.26 μm along the a-axis, increased from 1.45 μm to 1.52 μm along the *b*-axis, and increased from 3.28 μm to 6.12 μm along the c-axis. These results demonstrate that in seed-mediated systems, substantial KHSO_4_ addition drives preferential crystal growth along the [001] direction. Mechanistically, excess SO_4_^2−^ ions form hydrogen bonds with surface silanol groups, facilitating their selective attachment to the [001] crystal plane. This interaction reduces the activation energy barrier for [001]-oriented growth, enabling anisotropic crystal development [28,29]. This confirms KHSO_4_’s ability to directionally control zeolite crystal growth in seed-containing systems through facet-specific templating effects.

In terms of pore structure, the addition of KHSO_4_ first promoted grain aggregation to form intercrystalline pores, leading to a gradual increase in the specific surface area (S_BET_) for S1-0-0.24-2.42 wt% (186.10 m^2^/g), S1-0.05-0.24-2.42 wt% (212.07 m^2^/g), and S1-0.1-0.24-2.42 wt% (258.53 m^2^/g). The decrease in S_BET_ for S1-0.25-0.24-2.42 wt% (228.07 m^2^/g) was due to the influence of pH changes on zeolite crystallization. The increase in total pore volume (V_total_) (Table 1) was attributed to the formation of intercrystalline pores and the role of K^+^ [25].

#### 3.3.3. Investigation on Effects of K^+^ and SO_4_^2−^

Figure S2 presents the XRD patterns of Silicalite-1 synthesized with different salts as additives. All samples display characteristic diffraction peaks of the MFI structure, and their relative crystallinity values are comparable to that of sample S1-0.25-0.24-2.42 wt%. This indicates that the seed suspension still dominates the promotion of nucleation and crystallization in these systems containing various additives.

Distinct differences are observed among the samples: characteristic peaks of SiO_2_ appear in all samples doped with potassium salts, while no such peaks are detected for samples with sulfate additives. The results reveal that SO_4_^2−^ is the key species for suppressing the formation of impurity crystalline phases, and the presence of K^+^ does not interfere with this effect.

Figure S3 shows the SEM images of zeolites synthesized using different additives. Samples S1-KHCO_3_, S1-KNO_3_, S1-KCl and S1-KF all exhibit tetragonal crystal grains (Figure S4a,b), whose morphology is similar to that obtained using seed suspension alone. By contrast, samples synthesized with sulfate salts show plate-like crystals (Figure S3e–h). It is confirmed that the synergistic effect originates from the interaction between seed crystals and sulfate ions SO_4_^2−^. Sulfate ions selectively adsorb on specific crystal planes of Silicalite-1, inducing preferential crystal growth along the c-axis and ultimately forming anisotropic plate-like morphology. Meanwhile, the seed crystals provide a favorable synthesis environment that enables sulfate ions to fully exert their effects.

#### 3.3.4. Crystallization Process

In order to further understand the synergistic effect of KHSO_4_ and seeds on the growth of Silicalite-1 in very low organic template systems, we tracked the crystal growth of zeolite by controlling different synthesis times under hydrothermal conditions. The gel composition was maintained as 1SiO_2_:0.007TPABr:0.1KHSO_4_:0.24NaOH:13.66H_2_O:2.42 wt% seeds.

**Figure 8:**
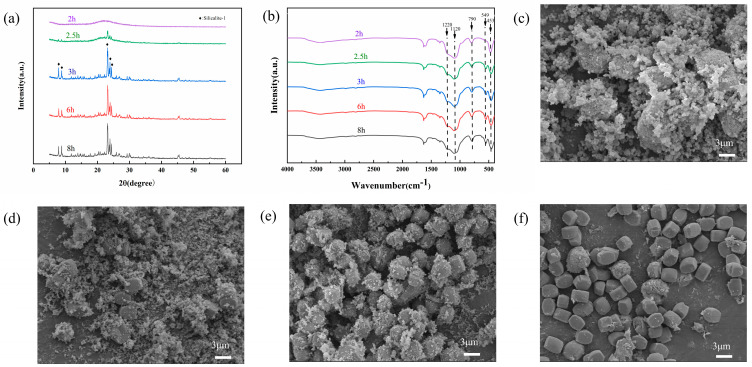
Synthesis of KHSO_4_ and seeds with different reaction times: (**a**) XRD; (**b**) FT-IR; SEM of (**c**) 2 h; (**d**) 2.5 h; (**e**) 6 h; (**f**) 8 h.

The XRD patterns, FT-IR, and SEM images of the samples are shown in Figure 8. It can be concluded that the crystallization process under this system is similar to that under the single seed system (Figure 2a,b and Figure 8a,b), indicating that the nucleation and growth of crystals are mainly controlled by seeds, while the role of KHSO_4_ is reflected in the crystal state. Different from the single seed system, large agglomerates can be observed in the SEM images at the initial stage of crystallization (2 h) (Figure 8c), which are formed by the polycondensation of silicates due to the presence of KHSO_4_, and then the crystals gradually grow to completion with the prolongation of crystallization time.

Combined with the results of scanning electron microscopy (SEM) images of S1-0.1-4.21 wt% (Figure 6g) and S1-0.25-2.42 wt% (Figure 7g), the synergistic mechanism of seed crystals and potassium bisulfate (KHSO_4_) on zeolite growth can be postulated as follows: In the sole seed suspension system, the silicon source undergoes hydrolysis and polycondensation reactions in an alkaline environment and rearranges to form crystal nuclei under the induction of the seed suspension, followed by the further growth of crystal nuclei into crystals [18,19,20,21,22]. The introduction of sulfate ions not only accelerates the polycondensation process, but can also be selectively adsorbed onto specific crystal facets through hydrogen bonding interactions, thereby achieving the regulation of crystal morphology [32,33]. The difference in the addition ratio of the two will significantly affect the properties of the final product.

**Scheme 1:**
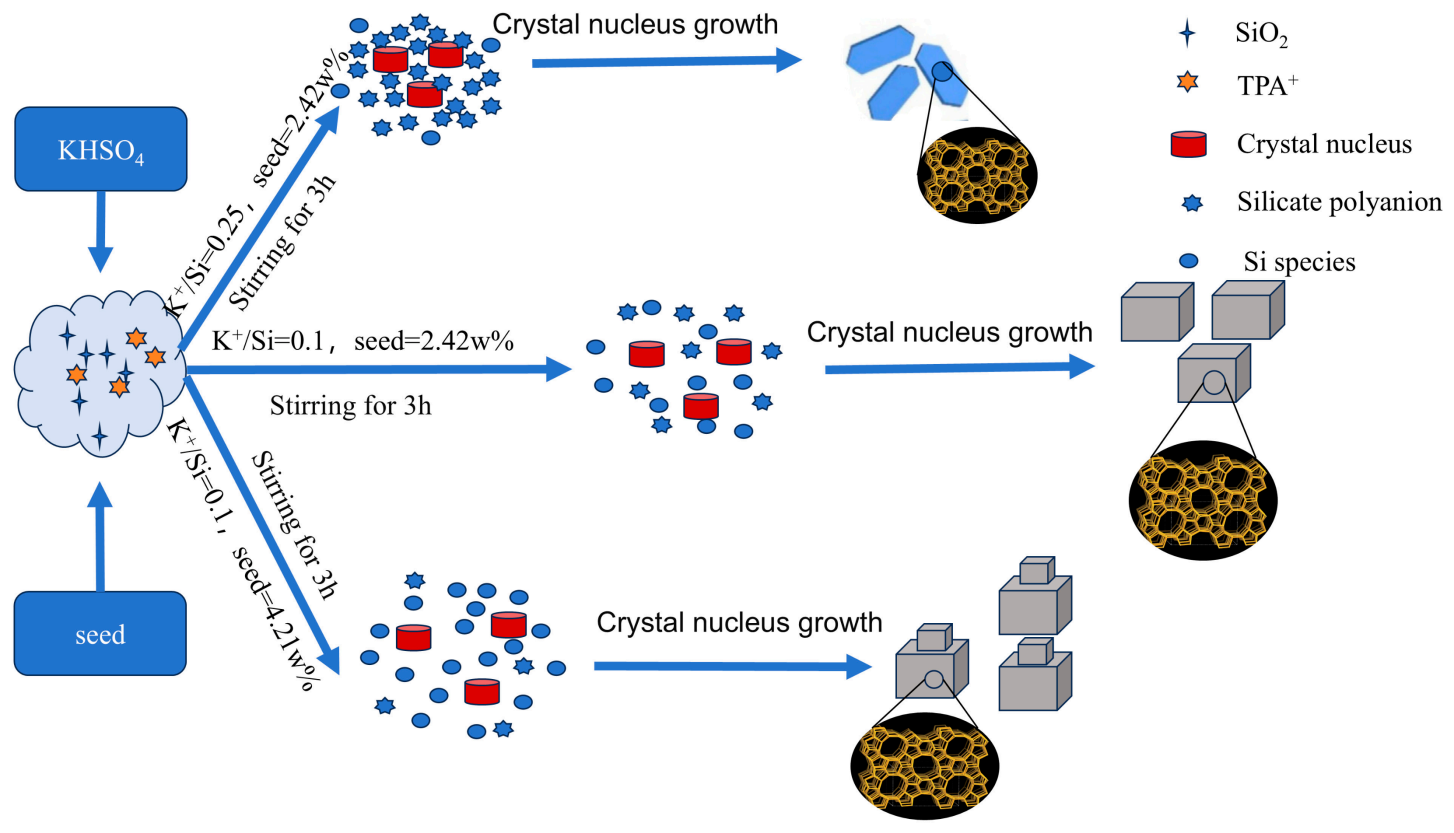
Schematic illustration of effects of seed and KHSO_4_ dosages on Silicalite-1 crystals.

When the content of potassium bisulfate is relatively high (x = 0.25, z = 2.42 wt%), a large number of sulfate ions are adsorbed on the [010] crystal plane through hydrogen bonding interactions, dominating the crystal growth orientation. Moreover, the strong effect of hydrogen bonds induces crystal agglomeration, and the crystals ultimately develop into plate-like crystals in a stacked and aggregated form. When the seed content is relatively high (x = 0.1, z = 4.21 wt%), an insufficient amount of sulfate ions can be adsorbed onto the crystal plane, leading to weak hydrogen bonding interactions. Under this condition, the crystal growth process is dominated and regulated by the seed crystals, eventually yielding weight-like crystals. When the contents of seed crystals and potassium bisulfate are comparable (x = 0.1, z = 2.42 wt%), the two components exert a mutual restrictive effect on each other, ultimately facilitating the formation of tetragonal crystals. Scheme 1 illustrates the crystal growth process under the coexistence of the two components.

**Scheme 2:**
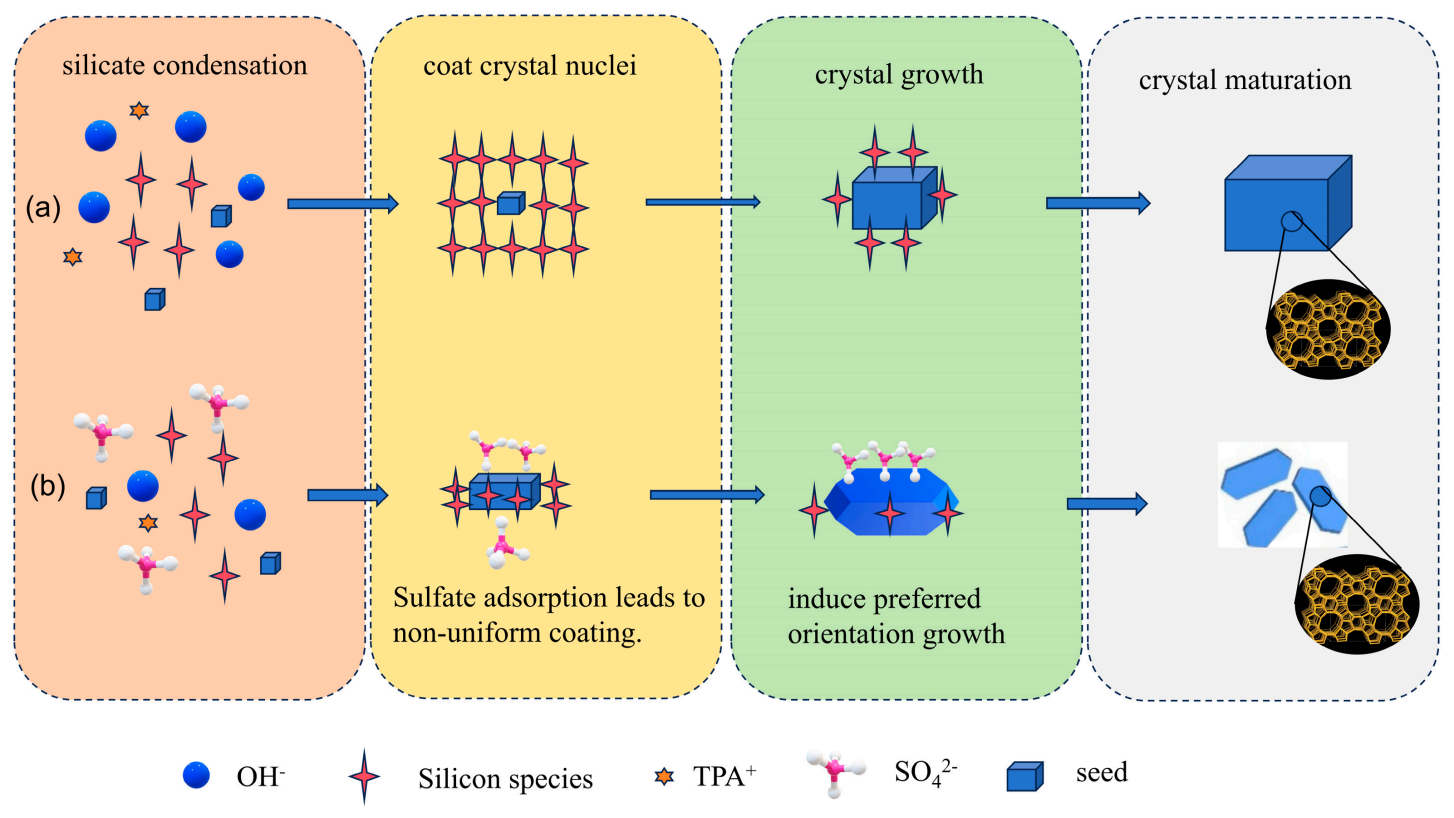
(**a**) Mechanism of seed induction without sulfate ions; (**b**) seed-induced mechanism in presence of SO_4_^2−^.

Based on the above analysis, it can be inferred that the crystallization in this system is governed by seed crystals and follows the non-classical crystallization mechanism. At the early stage of hydrothermal treatment, numerous amorphous nanoprecursors composed of silicon species form and attach to the surface of crystal grains. As crystallization proceeds, these metastable silicon-based nanoparticles gradually dissolve. The dissociated silicon oligomers continuously participate in framework construction, which optimizes the crystal structure and reduces surface deposits. At the late stage of crystallization, only a small number of unreacted particles remain, indicating that the non-classical growth process dominated by nanoprecursor attachment, dissolution and recrystallization is nearly completed, with only a minor contribution from conventional crystal growth via monomer addition. Sulfate ions interact with silanol groups through hydrogen bonding to affect the depolymerization of silicon species, and adsorb on specific crystal facets to guide preferential crystal growth. Notably, sulfate ions do not incorporate into the zeolite framework (Table S1). The mechanism of seed induction in the pure system and that in the presence of sulfate ions is illustrated in Scheme 2.

### 3.4. Adsorption Experiment

**Figure 9:**
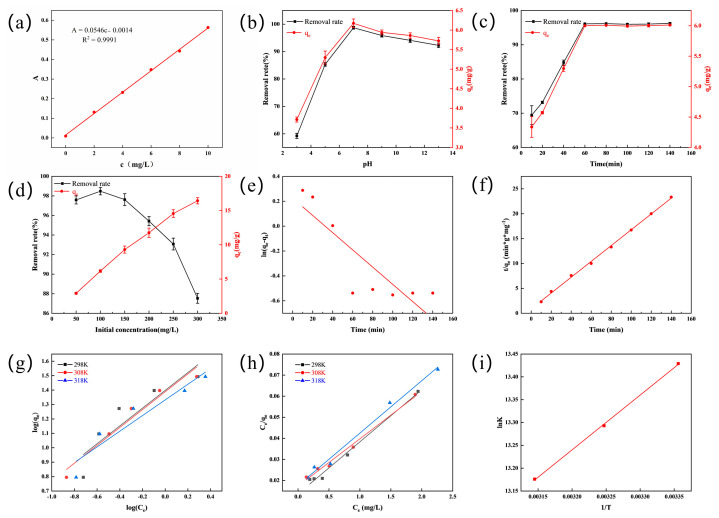
(**a**) Standard curve, (**b**) effect of pH value, (**c**) effect of adsorption time, (**d**) effect of initial solution concentration, (**e**) pseudo-first-order kinetic model, (**f**) pseudo-second-order kinetic model, (**g**) fitting plot of Freundlich adsorption isotherm model, (**h**) fitting plot of Langmuir adsorption isotherm model, and (**i**) thermodynamic plot.

As shown in Figure 9a, the zeolite sample S1-0.25-0.24-2.42 wt% was used as the adsorbent for methylene blue (MB). The absorbance of MB standard solutions was measured via ultraviolet-visible (UV-Vis) spectrophotometer at a wavelength of 664 nm. A standard curve of MB was plotted with absorbance as the ordinate and concentration as the abscissa. Linear regression yielded the equation A = 0.0546c − 0.0014 with a correlation coefficient R^2^ = 0.9991, indicating a good linear correlation.

The effects of pH, adsorption time and initial solution concentration were systematically investigated. The highest removal efficiency of MB was achieved at pH = 7. Under acidic conditions, competitive interaction between H^+^ and MB cations hinders adsorption, while excessively high pH also deteriorates the adsorption performance (Figure 9b). As the adsorption time increased from 10 min to 140 min (Figure 9c), the MB removal rate and equilibrium adsorption capacity rose continuously and then leveled off. This phenomenon is attributed to the coexistence of adsorption and desorption during the process. At the initial stage, the adsorption rate was higher than the desorption rate. As active sites on the adsorbent surface were gradually occupied, the desorption rate increased until the two rates became equal, reaching adsorption equilibrium. With the rise in initial MB concentration (Figure 9d), the adsorption capacity increased gradually, whereas the removal rate decreased. This is because the adsorbent possesses a limited number of active sites and cannot accommodate excessive solute when its dosage remains constant.

The adsorption kinetics of methylene blue onto the zeolite were investigated using the pseudo-first-order and pseudo-second-order models. The relevant results are presented in Figure 9e,f and Table S3. The correlation coefficients of the pseudo-first-order and pseudo-second-order models were 0.7062 and 0.9984, respectively. The pseudo-second-order kinetic model well described the adsorption behavior, demonstrating that the adsorption of methylene blue on sample S1-0.25-0.24-2.42 wt% was dominated by chemisorption [34].

Adsorption isotherms were further analyzed via the Freundlich and Langmuir models, as shown in Figure 9g,h and Table S4. Comparative analysis revealed that the Langmuir model achieved a superior fitting effect. Combined with the thermodynamic curves in Figure 9i, the enthalpy change ΔH° = −10.06 kJ/mol was a small negative value, indicating an energy-releasing process accompanied by typical physisorption characteristics. The positive entropy change ΔS° = +77.36 J/(mol·K) implied an increase in the disorder of the system. This phenomenon can be explained by the release of bound water molecules when methylene blue trihydrate molecules were adsorbed from aqueous solution onto the solid surface; the enhanced mobility of free water molecules led to entropy growth. The obvious entropy increase further confirmed that the adsorption was mainly governed by hydrophobic interaction.

The Gibbs free energy change ΔG° was negative at all tested temperatures, ranging from −33.27 kJ/mol to −34.84 kJ/mol (Table S5), which suggested that the adsorption occurred spontaneously within the investigated temperature range. The absolute value of ΔG° increased with rising temperature, proving that higher temperatures facilitated the adsorption process. According to Equation (S7) in the Supplementary Materials, the adsorption driving force originated from the combined effects of enthalpy change and entropy change, among which entropy change played a predominant role.

Based on the kinetic and thermodynamic fitting results, it can be inferred that the adsorption of methylene blue trihydrate on the zeolite is an entropy-driven process involving weak chemical interactions and specific physical interactions (hydrophobic effect). Kinetically, the process is controlled by surface active sites, while thermodynamically, it exhibits mild exothermic behavior and a remarkable entropy increase.

The regeneration performance of sample S1-0.25-0.24-2.42 wt% was evaluated in this work. As shown in Figure S4a, the methylene blue removal rate remained above 96% after five adsorption-regeneration cycles. This result indicates that the as-prepared sample possesses excellent reusability and can serve as a cost-effective adsorbent.

Figure S4b compares the adsorption capacities of S1-0.25-0.24-2.42 wt%, the scaled-up sample Su-S1-0.25-0.24-2.42 wt% and commercial zeolite (C-S1). The laboratory-synthesized sample and its scaled-up counterpart exhibited comparable removal efficiencies, both of which outperformed the commercial product. This further demonstrates the great industrial application potential of the synthetic strategy proposed in this study.

### 3.5. Scaled-Up Synthesis of Silicalite-1

In this work, scaled-up synthesis was carried out with the optimized molar ratio of 1SiO_2_:0.007TPABr:0.1KHSO_4_:0.24NaOH:13.66H_2_O:2.42 wt% seeds at a 20-fold amplification scale to verify the industrial applicability of the proposed synthesis route.

As shown in Figure S5a,b, the scaled-up product still retains the typical MFI structure. Its relative crystallinity is 81.31% (Table S2), which is close to 84.80% of the laboratory-scale sample. Plate-like crystals can also be observed in the SEM images. In addition, the scaled-up sample Su-S1-0.25-0.24-2.42 wt% exhibits the same adsorption capacity for methylene blue as the laboratory sample. These results demonstrate the success of the scaled-up synthesis of Silicalite-1 and further verify the industrial application potential of the synthetic strategy developed in this work.

## 4. Conclusions

In this study, a synergistic strategy combining a seed suspension and KHSO_4_ was developed for the controlled synthesis of Silicalite-1 zeolites under very low organic template conditions (TPA^+^/Si = 0.007). The seed suspension provides abundant structural units that significantly lower the nucleation barrier, accelerate crystallization, and reduce crystal size, while KHSO_4_ promotes the polycondensation of silicate species and effectively suppresses the formation of non-MFI impurity phases, such as cristobalite and quartz. More importantly, sulfate ions (SO_4_^2−^) selectively adsorb onto the specific crystal plane through hydrogen bonding interactions, directing preferential crystal growth along the c-axis. By adjusting the dosages of seeds (0–4.21 wt%) and KHSO_4_ (K^+^/Si = 0–0.25), the c-axis length of Silicalite-1 crystals can be precisely tuned, yielding either tetrahedron or plate-like morphologies. The crystallization follows a non-classical mechanism dominated by nanoparticle attachment, dissolution, and recrystallization, with sulfate ions guiding anisotropic growth. Scale-up synthesis in a 2 L autoclave confirms the industrial feasibility of this approach. The as-prepared zeolite exhibits excellent performance in methylene blue adsorption, with high removal efficiency (>96% after five cycles) and good reusability, outperforming a commercial counterpart. This work provides a low-cost, environmentally friendly, and scalable route for morphology-controlled synthesis of MFI-type zeolites, with broad implications for catalysis, separation, and wastewater treatment.

## Figures and Tables

**Table 1:** N_2_ adsorption–desorption and XRD parameters of Silicalite-1 samples.

Sample	Surface Area (m^2^/g)			Volume (cm^3^/g)			RC (%)	SRV (%)
	S_BET_	S_micro_	S_ext_	V_total_	V_micro_	V_meso_		
S1-0-0.24-0	98.84	88.80	10.04	0.13	0.04	0.09	41.36	100.00
S1-0-0.24-1.08 wt%	157.37	137.12	20.25	0.14	0.06	0.08	74.43	88.67
S1-0-0.24-2.42 wt%	186.10	173.65	12.45	0.15	0.08	0.07	90.09	54.29
S1-0-0.24-4.21 wt%	302.30	291.97	10.33	0.19	0.14	0.05	98.81	36.17
S1-0.05-0.24-0	117.88	85.88	32.00	0.18	0.04	0.14	52.18	41.69
S1-0.1-0.24-0	113.42	84.12	29.30	0.14	0.04	0.10	61.78	0
S1-0.15-0.24-0	87.52	60.00	27.52	0.11	0.03	0.08	55.91	0
S1-0.1-0.19-0	94.56	64.72	29.84	0.12	0.03	0.09	33.58	0
S1-0.1-0.29-0	130.82	79.62	51.20	0.21	0.03	0.18	54.55	37.05
S1-0.1-0.24-1.08 wt%	179.89	145.95	33.94	0.15	0.06	0.09	76.00	22.20
S1-0.1-0.24-4.21 wt%	299.74	275.57	14.17	0.22	0.13	0.09	100.00	0
S1-0.05-0.24-2.42 wt%	212.07	202.28	9.79	0.14	0.09	0.05	90.56	39.78
S1-0.1-0.24-2.42 wt%	258.53	227.11	31.42	0.19	0.10	0.09	91.63	0
S1-0.25-0.24-2.42 wt%	228.07	203.19	24.88	0.20	0.09	0.11	84.80	0

## Data Availability

The original contributions presented in this study are included in the article/Supplementary Materials. Further inquiries can be directed to the corresponding author.

## Supplementary Materials

The following supporting information can be downloaded at: https://www.mdpi.com/article/10.3390/ma19122634/s1, Figure S1: (a) XRD patterns of seed crystal suspension; (b) FT-IR spectra; Figure S2: XRD patterns of Silicalite-1 zeolite synthesized with different potassium salts and sulfates; Figure S3: SEM images of Silicalite-1 zeolite synthesized with different potassium salts and sulfates: (a) S1-KHCO_3_; (b) S1-KNO_3_; (c) S1-KCl; (d) S1-KF; (e) S1-NaHSO_4_; (f) S1-NH_4_HSO_4_; (g) S1-MgSO_4_; (h) S1-CaSO_4_; Figure S4: (a) Adsorption-regeneration cycles; (b) Comparison of methylene blue adsorption capacity of different samples; Figure S5: (a) X-ray diffraction patterns of sample Su-S1-0.25-0.24-2.42 wt%; (b) Scanning electron microscopy images; Table S1: EDS elemental compositions of samples: (a) S1-0-0.024-0, (b) S1-0-0.24-2.42 wt%, (c) S1-0.1-0.24-0, (d) S1-0-0.25-2.42 wt%, (e) S1-0.1-0.24-4.21 wt%; Table S2: RC and SRV values of various samples; Table S3: Fitting parameters of adsorption kinetic models; Table S4: Fitting parameters of the Langmuir and Freundlich models; Table S5: Thermodynamic parameters.
