# Supplementary material for: Controllable Synthesis of Silicalite-1 with Tailored c-Axis Length via KHSO4 and Seed Co-Additive Strategy
Source: Materials (Basel). 2026 Jun 18;19(12):2634. doi: 10.3390/ma19122634 (PMC13303832; doi:10.3390/ma19122634)
Supplement: Supplementary file 1 [file materials-19-02634-s001.zip › materials-4349157-supplementary.pdf]

Supplementary Materials

Controllable Synthesis of Silicalite-1 with Tailored c-Axis Length via $KHSO_4$ and Seed Co-Additive Strategy

Xiaojing Meng *, Liangxu Zhou, Junwei Huang and Min Li

College of Chemistry and Chemical Engineering, Chongqing University of Science and Technology,
Chongqing 401331, China; 2023205045@cqust.edu.cn (L.Z.); 2025206058@cqust.edu.cn (J.H.); limin1406@163.com (M.L.)

2. Materials and Methods

2.1 Materials

We used potassium fluoride (KF, AR, Shanghai Macklin Biochemical Co., Ltd.), potassium chloride (KCl, AR, Chengdu Kelong Chemical Reagent Factory), potassium bicarbonate ($KHCO_3$, AR, Shanghai Macklin Biochemical Co., Ltd.), potassium nitrate (KNO_3, AR, Shanghai Macklin Biochemical Co., Ltd.), sodium bisulfate ($NaHSO_4$, AR, Chengdu Kelong Chemicals Co., Ltd.), ammonium bisulfate (NH_4HSO_4, AR, Shanghai Macklin Biochemical Co., Ltd.), anhydrous calcium sulfate ($CaSO_4$, AR, Shanghai Macklin Reagents), and anhydrous magnesium sulfate ($MgSO_4$, AR, Tianjin Guangfu Fine Chemical Research Institute). Methylene blue trihydrate ($C_{16}H_{18}ClN_3S\cdot 3H_2O$, AR) was purchased from Shanghai Macklin Biochemical Co., Ltd. All chemicals and reagents were used as received without further purification. Commercial C-S1 zeolite used for comparison was obtained from Qingdao Yuanke Catalyst Co., Ltd.

.

2.2 Methods

2.2.1 Scale-up experiment

The scale-up synthesis was carried out in a 2 L Teflon-lined stainless steel autoclave. The gel had a molar composition of 1 SiO_2: 0.007 TPAOH: 0.25 $KHSO_4$: 13.64 H_2O : 0.24 NaOH, with 2.42 wt% seeds (relative to the total gel mass). The scale-up factor was 20. The detailed procedure was as follows. First, 734 g of water was added into a beaker, followed by the addition of 530 g of silica sol and 6.6 g of tetrapropylammonium bromide (TPABr). The mixture was stirred at room temperature for 10 min. Then, 35.74 g of seeds and 120 g of potassium bisulfate were added, and stirring was continued for another 10 min to obtain solution C. Separately, 33.4 g of sodium hydroxide was dissolved in 133.4 g of water, and the solution was cooled to

room temperature to give solution D. Under continuous stirring, solution D was slowly added dropwise to solution C to form a synthesis gel. The gel was stirred at room temperature for 3 h, then transferred into the Teflon-lined stainless steel autoclave and heated at 180 °C for 8 h. After the reaction, the solid product was collected by filtration, washed with deionized water until neutral, then dried at 120 °C for 3 h, and finally calcined at 550 °C for 5 h to remove the template. The sample was designated as Su-S1-0.25-0.24-2.42 wt%.

2.2.2 Synthesis with other additives

Comparative experiments were conducted with different additives. The molar composition of the gel was 1 SiO_2: 0.007 TPAOH: 0.25 additive : 13.64 H_2O : 0.24 NaOH, containing 2.42 wt% seeds. The experimental procedure was identical to that described in Section 2.2.2. The obtained samples were denoted as S1-additive.

2.2.3 Adsorption test for methylene blue wastewater

To evaluate the adsorption performance of the as-prepared sample S1-0.25-0.24-2.42 wt% toward methylene blue (MB), simulated wastewater was prepared using an aqueous solution of methylene blue trihydrate. The adsorption experiments were conducted in 250 mL Erlenmeyer flasks. In each flask, 50 mL of MB solution with varying initial concentrations was added, along with a certain amount of adsorbent. The mixture was then shaken at 125 r/min in a thermostatic shaker at the desired temperature. After the adsorption reached equilibrium, the MB solution was filtered through a 0.22 μm disposable filter membrane. An aliquot of 1 mL of the filtrate was transferred to a colorimetric tube, diluted to the mark, and mixed thoroughly. After standing for 10 min, the absorbance was measured using a spectrophotometer at a wavelength of 664 nm. The residual concentration of methylene blue in the solution was calculated based on the calibrated linear standard curve fitting equation. The removal efficiency was calculated by Equation (S1), and the adsorption capacity was calculated by Equation (S2).

$$q_e = \frac{(C_0 - C_e) \times V}{m} \quad \text{(S1)}$$

$$\eta = \frac{C_0 - C_e}{C_0} \times 100\% \quad \text{(S2)}$$

q_e: equilibrium adsorption capacity of methylene blue (mg/g); C_0: initial concentration of the solution (mg/L); C_e: equilibrium concentration of the solution (mg/L); m: mass of adsorbent (g); V: volume of solution (L); η: removal efficiency (%).

The obtained data were fitted with kinetic models, adsorption isotherm models and thermodynamic equations to analyze the adsorption behavior of methylene blue on the zeolite.

Kinetic models (pseudo-first-order kinetic model (S3) and pseudo-second-order kinetic model (S4)):

$$ln(q_e - q_t) = lnq_e - K_1 t \tag{S3}$$

$$\frac{t}{q_t} = \frac{1}{K_2 q_e^2} - \frac{t}{q_e} \tag{S4}$$

q_e: equilibrium adsorption capacity (mg/g); q_t: adsorption capacity at time t (mg/g); t: adsorption time (min); K_1: rate constant of pseudo-first-order kinetics; K_2: rate constant of pseudo-second-order kinetics.

Adsorption isotherm models: Langmuir equation (S5) and Freundlich equation (S6):

$$\frac{C_e}{q_e} = \frac{1}{q_m K_L} + \frac{C_e}{q_m} \tag{S5}$$

$$lnq_e = lnK_f + \frac{1}{n} lnC_e \tag{S6}$$

C_e: equilibrium concentration of methylene blue in solution (mg/L); q_m: maximum adsorption capacity of the adsorbent (mg/g); K_L: adsorption equilibrium constant related to adsorption heat (L/mg); K_f: constant related to adsorption capacity (L/mg); n: adsorption constant of the Freundlich isotherm.

Thermodynamic fitting equations:

$$\Delta G = \Delta H - T\Delta S \tag{S7}$$

$$lnK_c = \frac{\Delta S}{R} - \frac{\Delta H}{RT} \tag{S8}$$

$$K_C = \frac{q_e}{C_e} \tag{S9}$$

ΔG: Gibbs free energy (kJ/mol); R: universal gas constant (8.314 J/mol·K); K_c: Langmuir adsorption constant (L/mol); T: absolute temperature (K); ΔH: enthalpy change (kJ/mol); ΔS: entropy change (J/mol·K).

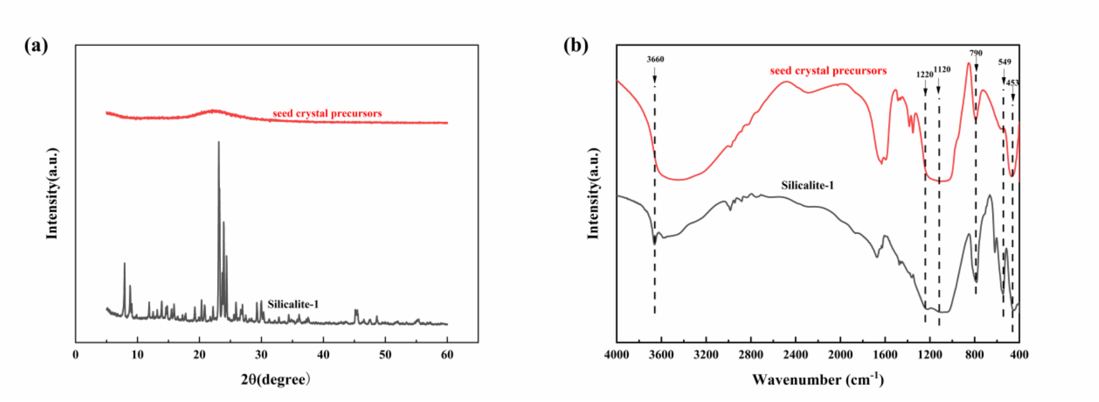

Figure S1. (a) XRD patterns of seed crystal suspension; (b) FT-IR spectra.

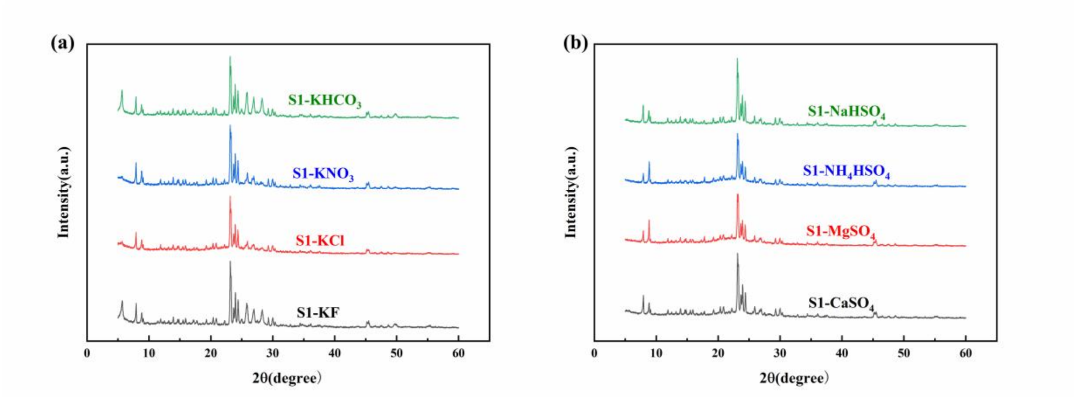

Figure S2. XRD patterns of Silicalite-1 zeolite synthesized with different potassium salts and sulfates.

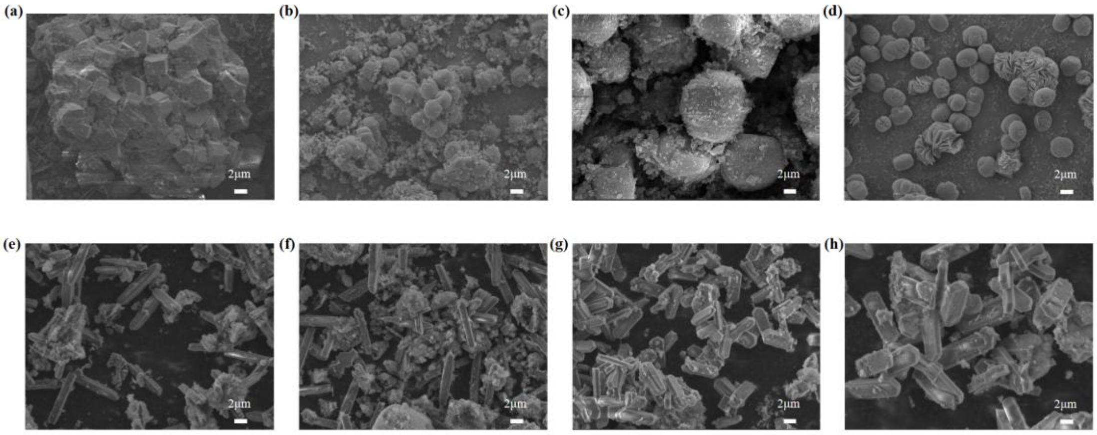

Figure S3. SEM images of Silicalite-1 zeolite synthesized with different potassium salts and sulfates: (a) S1-$KHCO_3$; (b) S1-KNO_3; (c) S1-KCl; (d) S1-KF; (e) S1-$NaHSO_4$; (f) S1-NH_4HSO_4; (g) S1-$MgSO_4$; (h) S1-$CaSO_4$.

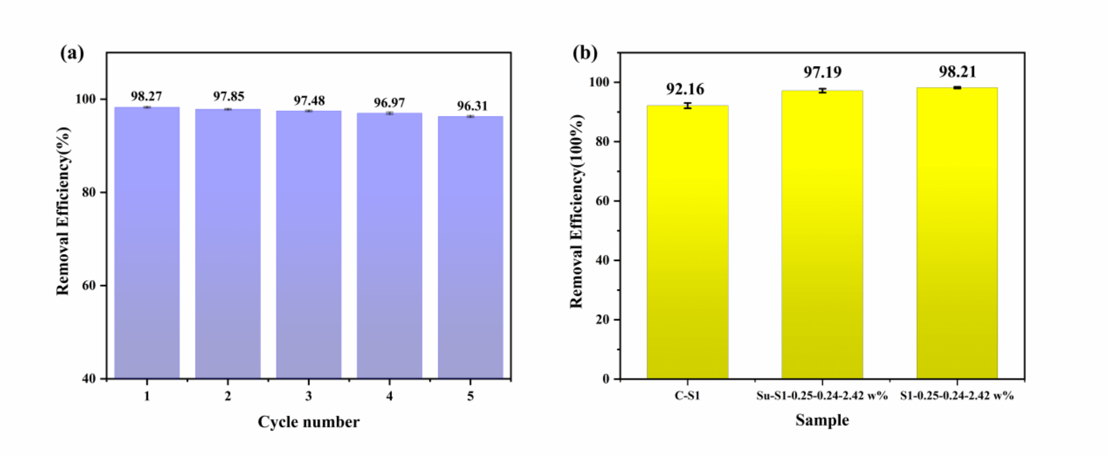

Figure S4. (a) Adsorption–regeneration cycles; (b) comparison of methylene blue adsorption capacity of different samples.

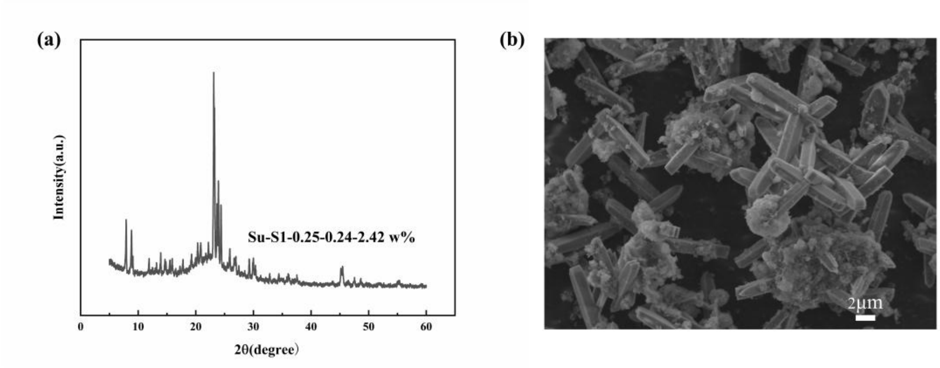

Figure S5. (a) X-ray diffraction patterns of sample Su-S1-0.25-0.24-2.42 wt%; (b) scanning electron microscopy images.

Table S1. EDS elemental compositions of samples: (a) S1-0-0.024-0, (b) S1-0-0.24-2.42 wt%, (c) S1-0.1-0.24-0, (d) S1-0-0.25-2.42 wt%, and (e) S1-0.1-0.24-4.21 wt%.

Sample		a		b		c		d		e	
		wt%	wt% Sigma	wt%	wt% Sigma	wt%	wt% Sigma	wt%	wt% Sigma	wt%	wt% Sigma
C	EDS	37.78	0.50	32.28	0.46	34.26	0.51	26.89	0.36	28.65	0.61
O	EDS	40.78	0.42	43.81	0.37	42.57	0.49	44.59	0.36	41.85	0.45
Na	EDS	1.03	0.07	1.15	0.07	-	-	8.16	0.11	0.75	0.08
Si	EDS	20.40	0.22	22.76	0.21	23.17	0.20	20.36	0.04	28.75	0.31
Total		100.00		100.00		100.00		100.00		100.00	

Table S2. RC and SRV values of various samples.

Sample	RC (%)	SRV (%)
S1-$KHCO_3$	76.47	87.53
S1-KNO_3	71.22	27.21
S1-KCl	79.28	23.17
S1-KF	74.21	53.31
S1-$NaHSO_4$	80.26	0
S1-NH_4HSO_4	69.23	0
S1-$MgSO_4$	75.26	0
S1-$CaSO_4$	78.38	0
Su-S1-0.25-0.24-2.42wt%	81.31	0

Table S3. Fitting parameters of adsorption kinetic models.

adsorption kinetic models	parameter	value
pseudo-first-order	R^2	0.70618
	$q_{e(exp)}$	6.29
	$q_{e(cal)}$	1.6778
	K_1	-0.517
pseudo-second-order	R^2	0.99839
	$q_{e(exp)}$	6.01
	$q_{e(cal)}$	6.29
	K_2	0.0289

Table S4. Fitting parameters of the Langmuir and Freundlich models.

	Langmuir			Freundlich		
T（K）	q_m(mg/g)	R^2	K_L	R^2	1/n	K_f
298	23.47	0.9899	1.8177	0.8215	0.6168	25.0034
308	17.80	0.9908	1.2994	0.9545	0.6188	24.4455
318	16.72	0.9906	1.4101	0.9055	0.5428	21.4728

Table S5. Thermodynamic parameters.

T（K）	ΔG（KJ/mol）	ΔH（KJ/mol）	ΔS（J/mol·K）	R^2
298	-33.27			
308	-33.53	-10.06	77.36	0.9994
318	-34.84			
